# Supplementary material for: Measurement of correlated charge noise in superconducting qubits at an underground facility
Source: Nat Commun. 2025 Nov 11;16:9906. doi: 10.1038/s41467-025-63724-4 (PMC12606239; doi:10.1038/s41467-025-63724-4)
Supplement: Supplementary file 1 — Supplementary Information [file 41467_2025_63724_MOESM1_ESM.pdf]

Supplementary Information for: Measurement of Correlated Charge Noise in Superconducting Qubits at an Underground Facility

G. Bratrud[2,1], S. Lewis[3,1], K. Anyang[4,1], A. Colón Cesaní[2], T. Dyson[5,6,7], H. Magoon[1,5,6,7,8], D. Sabhari[2], G. Spahn[9,1], G. Wagner[1], R. Gualtieri[2], N.A. Kurinsky[1,6,7], R. Linehan[1], R. McDermott[9], S. Sussman[1], D.J. Temples[1], S. Uemura[1], C. Bathurst[10], G. Cancelo[1], R. Chen[2], A. Chou[1], I. Hernandez[4,1], M. Hollister[1], L. Hsu[1], C. James[1], K. Kennard[2], R. Khatiwada[4,1], P. Lukens[1], V. Novati*[2], N. Raha[2], S. Ray[2], R. Ren†[2], A. Rodriguez[2], B. Schmidt‡[2], K. Stifter[1,6,7], J. Yu[4], D. Baxter[1,2], E. Figueroa-Feliciano[2,1], and D. Bowring §[1]

[1] *Fermi National Accelerator Laboratory, Batavia, IL 60510, USA*
[2] *Department of Physics & Astronomy, Northwestern University, Evanston, IL 60208, USA*
[3] *Department of Physics and Astronomy, Wellesley College, Wellesley, MA 02481, USA*
[4] *Department of Physics, Illinois Institute of Technology, Chicago, IL 60616, USA*
[5] *Department of Physics, Stanford University, Stanford, CA 94305, USA*
[6] *Kavli Institute for Particle Astrophysics and Cosmology, Stanford University, Stanford, CA 94305, USA*
[7] *SLAC National Accelerator Laboratory, Menlo Park, CA 94025, USA*
[8] *Department of Physics & Astronomy, Tufts University, Medford, MA 02155, USA*
[9] *Department of Physics, University of Wisconsin-Madison, Madison, WI 53706, USA*
[10] *Department of Physics, University of Florida, Gainesville, FL 32611, USA*

(Dated: August 26, 2025)

A Description of experimental apparatus

The qubit chip has an area of 6.25 x 6.25 mm^2. It incorporates four weakly charge-sensitive circular transmon qubits, as shown in Figure 2 of the main text. Each qubit consists of a circular superconducting Nb island set within a circular hole in the superconducting Nb groundplane (island radius $r_i = 70$ µm and groundplane inset radius $r_o = 90.5$ µm), with one Al/AlO$_x$/Al Josephson junction bridging the gap. For a uniform electric field, the sensing area of a single qubit is $\pi\epsilon r_i r_o$, with relative permittivity ϵ. Each qubit has a ratio of Josephson energy to single-electron charging energy $E_J/E_C = 24$ and is capacitively coupled to a charge bias control line (Figure 2, main text, orange) as well as a readout resonator (Figure 2, main text, yellow) for dispersive measurement through a shared feedline (Figure 2, main text, purple). The qubits are split into two pairs, one on each side of the central feedline, with center-to-center intra-pair separations of 340 µm and 640 µm, and inter-pair separation of around 3 mm. Additional qubit parameters are available in Table 1 in the main text, Supplementary Table 1, and in Ref. [1].

	Q1	Q2	Q3	Q4
res. (GHz)	6.1879	5.8283	6.0747	5.9593
f_{01} (GHz)	4.8336	4.7084	4.5318	4.6945
Δf_{01} (MHz)	2.6	3.1	3.9	3.4

Supplementary Table 1: **Measured frequencies of devices used.** Resonator frequency (res.) denotes the frequency of the readout mode, f_{01} is the qubit transition frequency, and Δf_{01} is the charge dispersion.

NEXUS is a low-background test stand for cryogenic detector calibration at Fermilab [2, 3]. The facility is located 107 m underground in the Neutrinos at the Main Injector (NuMI/MINOS) beam line access tunnel, with a rock and concrete overburden corresponding to 225 meters water equivalent [4]. With this overburden, the muon flux from cosmic rays is a factor of 200 lower than at a surface facility: approximately 7 muons cm^{-2} day^{-1}, with a negligible hadronic shower rate [5]. The Madison qubit package was installed in a Cryoconcept HEXA-DRY pulse-tube dilution refrigerator (DR) with passive vibration isolation and a modular lead shield that attenuates environmental radiation with 4π coverage. The DR and all experimental electronics operate in a class 10,000 clean room to minimize sources of particulate contamination and radioactivity from dust.

The NEXUS DR, shown in Supplementary Figure 1, is enclosed in a three-part lead shield: (1) a four-inch-thick inner lead plug above the payload, thermalized at $\sim$1 K; (2) a four-inch-thick stationary wall; and (3) a cart-mounted, movable lead shield with a nominal thickness of four inches. Taken to-

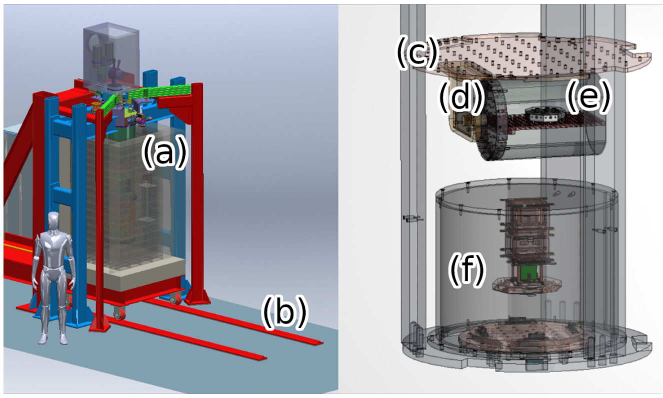

Supplementary Figure 1: **Schematic of NEXUS DR and shield assembly (left) and experimental payloads in the DR (right).** (a) The movable lead shield covers three sides of the DR. It rests on a wheeled platform. (The fourth side of the lead shield is permanently installed on the DR frame and platform, and is not visible in this schematic. (b) The shield platform rolls on a set of rails, permanently installed in the floor of the clean room. (c) NEXUS DR 10 mK plate. (d) A4K magnetic shield for qubit package. (e) Qubit package. (f) LMO detector package. The straight-line distance between the center of the qubit chip and the center of the LMO crystal is 18.7 cm. Cabling, instrumentation, thermalization, and other miscellaneous hardware and instrumentation are not shown.

gether, these three shield components provide full, 4π coverage of experimental payloads against ambient gamma radiation.

The qubit chip is installed inside of a 1-mm-thick A4K hermetic can from Amuneal, thermalized to the DR mixing chamber plate. The DR itself is further equipped with a 1-mm-thick A4K magnetic shield from Amuneal, thermalized at 4 K to reduce the magnetic field in the payload region. Finally, a external Metglass blanket surrounds the 300-K shell. The DR base temperature was stabilized at 10.5 mK during the collection of all data presented here.

B Electronics chain

A diagram of the electronics and DR stages is shown in Supplementary Figure 2. The qubit package has one input and one output connection to a common transmission line that feeds all four resonators. Each qubit has a separate connection for its charge bias line. All input lines consist of stainless steel coaxial cables from room temperature to 10 mK with attenuation and IR filters [6] to limit thermal loading. Output lines are NbTi superconducting coaxial cable between the 10 mK and 4 K stages to minimize losses and stainless steel from 4 K to room temperature. The output signals from the qubit package are amplified by a Josephson Traveling Wave Parametric Amplifier (labeled TWPA in Supplementary Figure 2) with a microwave pump tone at the 10 mK stage [7], and by a high-electron-mobility transistor (HEMT) amplifier at the 4 K stage.

Two different warm RF systems are used for qubit characterization and measurement. Both are connected to the qubit input and output ports via a warm RF switch (not shown in Supplementary Figure 2). A vector network analyzer (not shown in Supplementary Figure 2) is used for continuous wave measurements during initial qubit characterization. Pulsed RF measurements are performed using the Quantum Instrumentation and Control Kit (QICK): a Xilinx RFSoC board with custom open-source software and firmware for the control of qubit systems [8, 9]. The qubit charge bias voltage is supplied by an arbitrary waveform generator (AWG). The system has since been upgraded to allow for multiplexed simultaneous readout, but at the time of the measurement presented in the main text, this was not possible. As such, individual qubits are measured consecutively for each charge bias voltage in a Ramsey tomography scan before moving onto the next bias value.

C The response of layered superconducting devices to ionizing events

Consider the energies and time scales involved in the absorption and dissipation of ionizing radiation in a silicon substrate, layered with superconducting aluminum. Cosmic and gamma rays typically deposit hundreds of keV of energy in a chip. From the initial energy deposition, one electron-hole pair is generated per 3.7 eV deposited into the electronic system [10] along with a burst of phonons as energy is transferred to the crystal lattice. The electrons and holes can recombine promptly or after some diffusion, producing more phonons. Alternatively, they can be trapped by impurities in the substrate material. If not collected, these trapped charges are

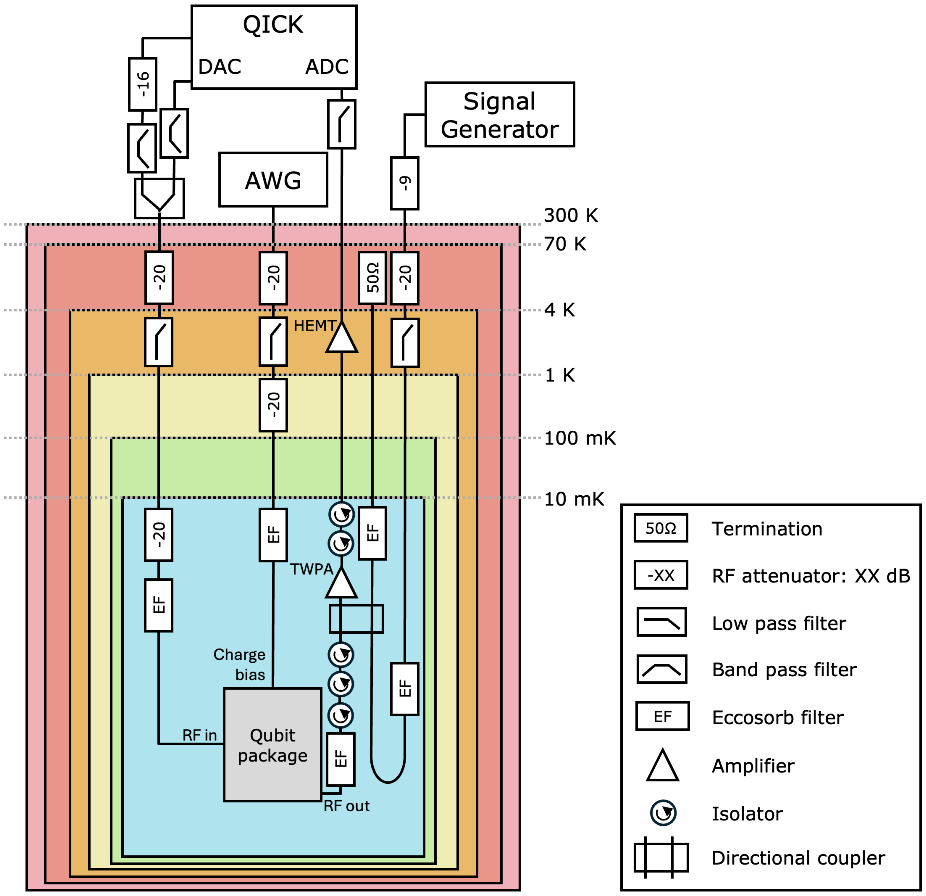

Supplementary Figure 2: **Block diagram of experimental apparatus.** The readout signals are carried on the QICK DAC line, with 16 dB of attenuation. The control pulses are carried on the other DAC line. There are four charge bias lines output from the AWG, one for each qubit. Each charge bias line is attenuated and filtered the same; only one line is shown in the diagram for visual clarity.

quasi-stable, with lifetimes in the range of hours to days at mK temperatures [11], and will alter the ambient electric field at the qubit island. The phonons, by comparison, become quasi-diffusive within 50 ns, then travel ballistically and potentially interact with the superconducting metal films which make up the qubit resonator structures at the crystal surfaces. At the superconducting film, these phonons are sufficiently energetic to break Cooper pairs, creating an excess population of Bogoliubov Quasiparticles (QPs). This excess QP population typically takes milliseconds to dissipate [12].

Straightforward measurements of T_1, typically taking tens of microseconds, are therefore fast relative to both QP dissipation and timescales on which trapped charge is released. While the QP population can be inferred from T_1 measurements [13], the effects of charge movement and recombination in the substrate have a nontrivial time structure. Accordingly, the focus in this work is on observing fluctuations in offset charge over many hours.

D Measurement Methodology

To track charge bursts over time we use the same Ramsey tomography procedure as in Ref. [1]: we apply a gate sequence $X/2$–*Idle*–$X/2$, followed by a readout pulse. During the idle time, the state vector acquires a phase that depends on n_g, as in Equation 1 in the main text. We choose the idle time to be $1/(4\Delta f_{01})$, where Δf_{01} is the maximum frequency separation of the two parity bands. The final $X/2$ pulse then maps the resulting state vector onto the $|1\rangle$ state and is probed via the readout resonator. We wait $10\,T_1$ before starting the next measurement, ensuring passive initialization of the qubit into the $|0\rangle$

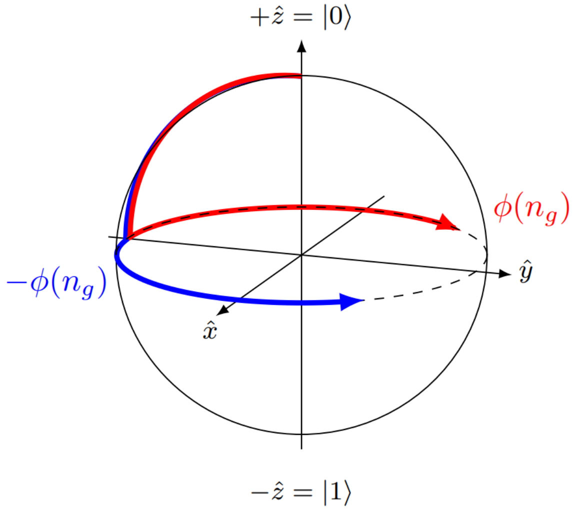

Supplementary Figure 3: **Bloch sphere illustrating charge tomography sequence.** Red and blue arrows indicate the parity-dependent path of the Bloch vector.

state. Our dephasing times T_2^* are measured from Ramsey experiments to be greater than $1\,\mu$s in all qubits, sufficiently long compared to our pulse timing so that we do not expect decoherence during a measurement.

This measurement sequence is averaged 200–300 times (depending on the qubit) for each applied charge bias voltage value. At each value, corresponding to an unknown overall value of n_g, measurements on each qubit are taken sequentially, meaning all 200 individual measurements are taken and averaged on qubit 1, then qubit 2, and so on, before the applied charge bias voltage value is changed and the measurement cycle is repeated. Each individual Ramsey sequence takes 0.0049 s. Acquiring full statistics at each charge bias point takes 0.98–1.479 s per qubit. One full tomographic scan across all charge bias points takes 355 s.

The time-correlation window of 44 s is limited by our data-acquisition methodology. Charge jumps in multiple qubits are considered correlated if they are situated within a 10-point window in a given tomography scan. Since each point takes 0.98 s for qubits 1-3, and 1.4 s for qubit 4, and since qubit readout is interleaved, two neighboring points for the same qubit are about 4.4 s apart in time. We chose a window of 10 points (44 s) for identifying correlated jumps due to the "time" (number of points) it takes for an average-size charge jump to be identified through our minimum combined χ^2 methodology.

E Jump-finding algorithm

We utilize a data-driven method to identify charge jumps: a template for each qubit averages together 15–23 scans from the S.C. datasets that are free of charge jumps larger than $0.03e$, the approximate detectable jump size given our specific averaging requirements. The fluctuations for each selected jump-free set are randomly distributed in time. (Qubit 4 required two templates, one for S.C. and one for S.O., due to reproducible noise differences between shield configurations.) Templates are one period long, with a full scan created by stitching together period templates to the required length. The error for each point in the template is derived from the standard deviation across all template scans at that charge bias point.

Once the template for each qubit is obtained, we find the best-fit phase θ and minimum reduced χ^2 from fitting the template to the first n points, for each value of n in $(1, N)$:

$$\chi_n^2(\theta) = \frac{1}{n}\sum_i^n \frac{(P_{1,i} - \hat{P}_1(\theta))^2}{\hat{\sigma}^2(\theta)}. \qquad (1)$$

for excited state probability P_1. The minimum value of $\chi_n^2(\theta)$ is calculated for each value of $n \in (1, N)$ where N is the total number of points in the scan, with the value θ_min^n corresponding to the phase associated with the minimum χ^2 at that point.

Charge jumps cause discontinuities in θ. If a discontinuity is present, the quantity $\chi_n^2(\theta_\mathrm{min})$ will rapidly begin to increase with each subsequent term in the sum. When the rolling χ^2 value exceeds a pre-set threshold (set individually for each qubit), a jump is identified and the procedure resets, with the first point over the threshold being reset as $i = 1$ in Supplementary Equation (1) and N limited to the remaining number of points in the scan. Because this is a cumulative process, it has limited efficiency in finding jumps in the first ~ 20 points of each scan. The minimum jump size resolution is qubit-dependent since each qubit has a slightly different relationship between DAC voltage and offset charge. This value varies from $0.026e$ to $0.029e$.

	Efficiency
Q1	0.83 ± 0.01
Q2	0.79 ± 0.01
Q3	0.87 ± 0.03
Q4 (S.C.)	0.72 ± 0.02
Q4 (S.O.)	0.74 ± 0.05

Supplementary Table 2: **Efficiencies to identify charge jumps.** Jupms with magnitude $0.1e \leq |\Delta q| \leq 0.5e$ are represented here. Errors represent systematic variation in the ability to find jumps of different sizes in this range.

The efficiency of this method is assessed by applying it to synthetic data with charge jumps inserted at known locations. For each qubit we simulate 1600 scans with a jump rate of 1.1 mHz (slightly higher than the actual rate to ensure we do not see effects of pile-up). The jumps are randomly injected with sizes selected from a flat probability distribution between $0.01e$ and $0.5e$. The simulated scans are produced using the templates discussed above and convolved with a Gaussian noise spectrum whose standard deviation is derived from errors at each template point. The accuracy of the jump-finding algorithm is calculated from the fraction of known charge jumps in the synthetic data that are correctly identified. Systematic errors on this quantity are derived from the standard deviation of this value for 15 different jump sizes across 75 sets of simulated scans per qubit (see Supplementary Table 2). This effectively captures the variation in efficiency across the range of $0.1e \leq |\Delta q| \leq 0.5e$, which can be crudely approximated by Gaussian (with slightly lower efficiency near the $0.1e$ threshold in particular).

The χ^2-threshold and the size of the low-efficiency window at the beginning of a scan, discussed above, are tuned to minimize false positives (e.g. noise tagged as jumps) while keeping the efficiency for tagging real jumps as high as possible. We calculate an efficiency for detecting real jumps of $> 70\%$ for $|\Delta q| > 0.1e$. This efficiency is fundamentally limited by the difficulty of finding jumps at the start and end of individual scans; improvements will be the subject of future work. Event rates in this data are sufficiently low that systematic uncertainties in our efficiency determination are subdominant to our statistical uncertainties. Errors presented in the main text are therefore Poissonian.

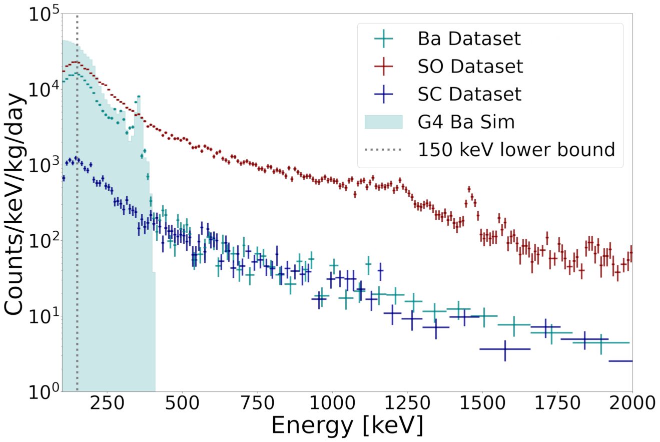

Supplementary Figure 4: **Energy spectra collected with a TES-coupled LMO detector.** Data collected in the S.O. configuration are shown in red, and S.C. configuration in blue. We acquired data with a ^{133}Ba source (teal) to validate our analysis and compared it to a Geant4 simulation (teal, shaded). The vertical grey line at 150 keV indicates the threshold above which the response of the LMO detector is unitary and above which the ratio of events measured in the S.O. to S.C. dataset is found to be 20 ± 1.

F Radiation background characterization

We characterize the spectrum of radiation incident on the qubit package through the use of another detector – a Li_2MoO_4 (LMO) crystal read out with a Transition-Edge Sensor (TES) [14]. This detector is a cryogenic calorimetric device that uses a 2-cm cube of LMO with a mass of 21 g. A 1 cm-diameter, 400-nm-thick gold film deposited on one of its sides through electron-beam evaporation. A TES calorimeter on a separate, $3 \times 3 \times 0.4$-mm Si chip is connected through a gold wire bond and measures the energy depositions from gammas in the LMO [15, 16]. The LMO and the qubit chip operate simultaneously inside the same DR. The LMO therefore enables a direct measurement of the gamma flux 18.7 cm away from the qubit chip. The energy spectrum for S.C. and S.O. data, as recorded by the LMO device, are shown in Supplementary Figure 4.

The integral of these spectra above 150 keV, in which range the LMO energy efficiency is well-characterized, is used to calculate the gamma flux ratio between S.O. to S.C.. This ratio is insensitive to systematic errors related to energy-independent

detector response, acceptance, and live-time effects and represents a direct measurement of the effect of the NEXUS shield on radiation flux. By closing the lead shield, the overall gamma flux is reduced in the LMO crystal by a factor of $A_{\mathrm{LMO}} = 20 \pm 1$ for energies above 150 keV. The energy scale in the S.O. data is calibrated using the ^{40}K peak at 1460 keV.

The qubit payload was exposed to ^{133}Ba and ^{137}Cs sources. The S.C. data does not have any peaks to use for calibration. To calibrate, we use data taken with a ^{133}Ba source inside the lead shield and compare with a `GEANT4` [17] simulation. The two ^{133}Ba and S.C. datasets were taken with the LMO device in the same bias conditions. The 356-keV peak is used to set the energy scale in the ^{133}Ba data, and the same energy calibration is used for the S.C. dataset. The `GEANT4` simulation only simulates the ^{133}Ba source, not the ambient S.C. spectrum. As shown in Supplementary Figure 4, the Ba dataset agrees very well with the S.C. dataset above 400 keV; above that rough threshold there is no flux from the ^{133}Ba source.

The lead shield was closed during the collection of this data, with the sources positioned inside the lead shield but outside the cryostat. The ^{133}Ba data, used to calibrate our LMO detector energy scale, had an integrated gamma rate close to the S.O. data (see Supplementary Figure 4). Operational issues at the underground facility limited our ^{133}Ba source exposure time to six hours, such that no statistically meaningful comparison can be made between this data set and the S.O. and S.C. data sets discussed in this paper. However, the measured jump rates at these low statistics are similar to the S.O. jump rates, as expected from a gamma-dominated scenario. Similarly, a ^{137}Cs source was installed to study the qubit response to higher gamma fluxes. This ^{137}Cs source saturated the LMO detector, making spectrum calibrations impossible in that configuration. The ^{133}Ba and ^{137}Cs data sets are therefore not included in this analysis. Future work will focus in more depth on the use of external gamma sources to vary the flux and spectrum of ionizing radiation on this qubit package.

At lower energies, the `GEANT4` simulation predicts a higher flux than we see in the LMO, which is likely a combination of the non-unity efficiency of the LMO at lower energies and an insufficiently detailed model in `GEANT4` of the material interposed between the ^{133}Ba source and the LMO. As mentioned above, by taking the ratio of S.O. and S.C. data we make a flux ratio which is insensitive to most of these systematic effects.

We exploit the measured ratio between the S.O. and S.C. LMO datasets A_{LMO} by solving for a constant excess charge jump rate R_{excess} present in both tomography datasets and the gamma-induced charge jump rate for each. By solving,

$$\begin{aligned} R^{SO} &= R_{\gamma}^{SO} + R_{\mathrm{excess}} \\ R^{SC} &= R_{\gamma}^{SC} + R_{\mathrm{excess}} \\ R^{SO} &= A_{\mathrm{LMO}} \times R_{\gamma}^{SC}, \end{aligned} \tag{2}$$

where $R^{SO/SC}$ is the measured charge jump rate in the S.O. and S.C. data and $R_{\gamma}^{SO/SC}$ is corrected gamma-induced rate in each dataset, we find an excess charge jump rate of $R_{\mathrm{excess}} = 0.17^{+0.04}_{-0.03}$. These results are presented in Table 1 of the main text.

References

[1] Wilen, C. D. *et al.* Correlated charge noise and relaxation errors in superconducting qubits. *Nature* **594**, 369–373 (2021).

[2] Temples, D. J. *et al.* Performance of a phonon-mediated kinetic inductance detector at the nexus cryogenic facility. *Physical Review Applied* **22**, 044045 (2024).

[3] Chen, R. *et al.* Modeling and characterization of TES-based detectors for the RICOCHET experiment. *Journal of Low Temperature Physics* (2024).

[4] Adamson, P. *et al.* Observation of seasonal variation of atmospheric multiple-muon events in the MINOS Near and Far Detectors. *Phys. Rev. D* **91**, 112006 (2015). URL `https://link.aps.org/doi/10.1103/PhysRevD.91.112006`.

[5] Garrison, L. *Measurement of Neutron and Muon Fluxes 100 m Underground with the SciBath Detector*. Ph.D. thesis, Indiana U., Bloomington (main) (2014).

[6] Spahn, G., Kurinsky, N., Lewis, S., Bowring, D. & Hollister, M. Epoxy IR filters for superconducting resonators. *Journal of Low Temperature Physics* **209**, 1032–1037 (2022).

[7] Macklin, C. *et al.* A near-quantum-limited Josephson traveling-wave parametric amplifier. *Science* **350**, 307–310 (2015).

[8] Ding, C. *et al.* Experimental advances with the QICK (Quantum Instrumentation Control Kit) for superconducting quantum hardware. *Physical Review Research* **6**, 013305 (2024). 2311.17171.

[9] Stefanazzi, L. *et al.* The QICK (Quantum Instrumentation Control Kit): Readout and control for qubits and detectors. *Review of Scientific Instruments* **93**, 044709 (2022).

[10] Ramanathan, K. & Kurinsky, N. Ionization yield in silicon for eV-scale electron-recoil processes. *Physical Review D* **102**, 063026 (2020).

[11] Kurinsky, N. A. *The Low-Mass Limit: Dark Matter Detectors with eV-Scale Energy Resolution.* Ph.D. thesis, Stanford University (2018). URL https://www.osti.gov/biblio/1472104.

[12] De Visser, P. *et al.* Number fluctuations of sparse quasiparticles in a superconductor. *Physical Review Letters* **106**, 167004 (2011).

[13] Catelani, G., Schoelkopf, R. J., Devoret, M. H. & Glazman, L. I. Relaxation and frequency shifts induced by quasiparticles in superconducting qubits. *Physical Review B* **84**, 064517 (2011).

[14] Bratrud, G. *et al.* First demonstration of a tes based cryogenic Li_2MoO_4 detector for neutrinoless double beta decay search. *The European Physical Journal C* **85**, 1–14 (2025).

[15] Chen, R., Pinckney, H. D., Figueroa-Feliciano, E., Hong, Z. & Schmidt, B. Transition edge sensor chip design of a modular CEνNS detector for the Ricochet experiment. *Journal of Low Temperature Physics* **211**, 237–247 (2023).

[16] Augier, C. *et al.* Results from a prototype TES detector for the RICOCHET experiment. *Nuclear Instrumentation Methods A* **1057**, 168765 (2023).

[17] Agostinelli, S. *et al.* Geant4—a simulation toolkit. *Nuclear Instrumentation Methods A* **506**, 250–303 (2003). URL https://www.sciencedirect.com/science/article/pii/S0168900203013688.
